# Targeting Internet Addiction Through Body Awareness Psychotherapy: The Mediating Role of Executive Functions and Emotion Regulation

**DOI:** 10.1002/brb3.70846

**Published:** 2025-09-02

**Authors:** Maliheh Fallah, Hamidreza Mohajerani, Abdullah Omidi, Amir Ghaderi

**Affiliations:** ^1^ Department of Addiction Studies, School of Medical, AND Clinical Research Development Unit‐Matini Kargarnejad Hospital, Kashan University of Medical Sciences Kashan Iran; ^2^ Quantum Technologies Research Center, Science and Research Branch Islamic Azad University Tehran Iran; ^3^ Department of Clinical Psychology, School of Medicine Kashan University of Medical Science Kashan Iran

**Keywords:** body awareness psychotherapy, cognitive function, emotion regulation, internet addiction, time perception

## Abstract

**Background:**

Internet addiction disorder (IAD) is increasingly recognized as a behavioral addiction linked to deficits in executive functioning, emotion regulation, boredom susceptibility, and impaired time perception. Emerging therapies such as body awareness psychotherapy (BAP) aim to improve cognitive and emotional self‐regulation through embodied mindfulness. This study aimed to assess the effectiveness of BAP in reducing internet addiction and related issues with time perception and boredom, focusing specifically on the mediating roles of executive functions and emotion regulation.

**Materials and Methods:**

A randomized controlled trial was conducted with 60 university students (aged 18–30) diagnosed with IAD. Participants were assigned to either the intervention group, which received BAP (*n* = 30), or the control group, which participated in general psychological discussions (*n* = 30). Over 8 weeks, the BAP group received structured training in body literacy, interoception, and self‐regulation. Assessments included Chen's Internet addiction scale, the multidimensional state boredom scale, the emotion regulation difficulties scale, the time perception task, the Stroop task, and the Iowa gambling task. Mediation analyses were performed using bootstrapped structural equation modeling.

**Results:**

BAP significantly reduced levels of internet addiction, boredom, and decision‐making deficits (*p* < 0.001). It also improved time perception and executive functions. Mediation analyses confirmed that improvements in executive functions—cognitive flexibility, impulse control, and response inhibition—and emotion regulation, significantly mediated the relationship between BAP and reduced internet addiction (*p* < 0.05).

**Conclusions:**

BAP is an effective intervention for reducing internet addiction and enhancing executive function and emotional regulation. Its efficacy is mediated by improvements in cognitive function, impulsivity, decision‐making, and emotion regulation, making it a promising tool for addressing behavioral addictions.

## Graphical abstract

Body awareness psychotherapy is an effective intervention for reducing internet addiction and enhancing executive function and emotional regulation. Its efficacy is mediated by improvements in cognitive function, impulsivity, decision‐making, and emotion regulation

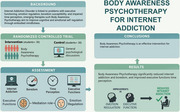

## 1 Introduction

In recent decades, social networks and the internet have gained significant popularity. New technological tools, such as smartphones, have become more essential than ever. Misuse of the Internet can lead to disruptions in individual well‐being (Bergquist et al. 2009; González‐Bueso et al. 2018) and can result in Internet addiction disorder (IAD). The prevalence of internet addiction has been reported variably in different studies. Recent European research has reported a prevalence range of 4.4%–13.5% for pathological internet use and 14.3%–54.9% for problematic internet use (Rabinovici et al. 2015; Verdejo‐Garcia et al. 2006).

On the other hand, executive functions refer to a set of cognitive skills that guide and enable adaptive, goal‐oriented behavior. Deficits in executive functioning can interfere with an individual's daily activities and routines, contributing to the development and persistence of IAD (Z. Zhou et al. 2016). Internet addiction is not just a behavioral addiction; it is also a disorder of impulse control, where impulsivity is seen as a vulnerability trait for both internet and pathological gambling addictions. Impulsivity is a crucial personality trait that can predict a person's lack of control over addictive behaviors and plays a vital role in addictive conduct. Another important feature of addiction, including internet addiction, is disinhibition; lack of inhibition raises vulnerability to developing addictive disorders and acts as a risk factor for maintaining such disorders while also contributing to negative emotional experiences (Khanbabaei et al. 2022; Rabinovici et al. 2015; Z. Zhou et al. 2016).

In IAD, distorted time perception is common and often linked to prefrontal dysfunction and attentional dysregulation, both hallmarks of impaired executive control. Time perception describes an individual's subjective experience of time and their interpretation of how long events last. Research indicates that internet addicts perceive time as passing more slowly than usual, possibly due to impulsivity (Khanbabaei et al. 2022; Kim et al. 2017; Paasche et al. 2019; Rabinovici et al. 2015; Z. Zhou et al. 2016). According to models, time perception depends on two distinct processes: a conscious, attentional process focused on time (prospective perception) and an unconscious process where attention is diverted from time (retrospective perception) (Ogden et al. 2011; Paasche et al. 2019; Turel and Cavagnaro 2019). Subjective time tends to stretch during boring activities like waiting or standing in line but passes quickly during enjoyable or exciting activities such as attending a party or watching a favorite show (Nuyens et al. 2019; Zhang et al. 2016).

Studies show that emotion regulation reduces the impact of emotional stimuli on time perception, playing a vital role in the interaction between cognitive skills and emotions (Farb et al. 2012; Hormes et al. 2014; Verdejo‐Garcia et al. 2006). Research has demonstrated a link between boredom and various psychological and social issues, such as impulsivity and reactivity, indicating that boredom influences both the mechanisms of boredom itself and addiction (Wang 2018). The two main components of boredom are limited stimulation and a uniform environment. When these conditions are present, the inability to attract and sustain attention typically results in boredom. Another important predictive factor for addictive behaviors is decision‐making. Decision‐making occurs in two stages before taking action, and addicts show deficits in both. The first stage involves assessing the value and utility of available options to determine priorities. The second stage is selecting an action and executing the decision (Bench and Lench 2013; Gjesme 2006; Y. Wang et al. 2019; Z. Wang et al. 2020). By enhancing interoceptive sensitivity and strengthening the neural networks responsible for self‐regulation and future‐oriented planning, Body Awareness Psychotherapy restores the somatic‐affective grounding necessary for adaptive decision‐making, especially under emotionally charged conditions that often trigger compulsive internet use (Aposhyan 2004; Bechara et al. 2002; Cole et al. 2004).

Recently, there has been a surge of interest in new research and methods for intervening in cognitive disorders related to addiction treatment. It has been shown that individuals with substance use disorders experience neuropsychological deficits in attention, memory, executive functions, language, and processing speed. This also applies to behavioral addictions, particularly internet addiction (Anderson et al. 2007; Noël et al. 2013; Sharma and Palanichamy 2018). New intervention models based on bidirectional feedback between the brain and body have emerged. These interventions utilize relatively simple muscle movements, rhythmic breathing exercises, aerobic activities, meditation, or combined mental and physical strategies (or cognitive enhancement) supported by their physiological nervous systems, influencing motivation regulation and stress resilience—all of which have shown effectiveness in managing severe chronic alcohol consumption and addiction (Li et al. 2020; Mischke‐Reeds 2018). Body compassion interventions, which incorporate mindfulness, emotional awareness, and self‐kindness, have been proposed to support this integration. Such interventions aim to reduce body image distress, improve emotional regulation, and promote overall well‐being in women after cancer (Sebri et al. 2023). Body awareness is a therapeutic approach that strengthens the mind‐body connection through mindfulness and interoception. It helps improve executive functioning and emotion regulation, making it effective for reducing compulsive behaviors associated with internet addiction (Aposhyan 2004).

Among these methods, body awareness is one intervention aimed at the brain‐body connection and emotional regulation. Body awareness training is based on a mind‐body approach, designed to teach body awareness skills, self‐care, and emotion regulation (Aposhyan 2004; Price and Smith‐Dijulio 2016). It includes processing sensory input from the body. Body awareness is a cognitive attitude focusing on physical symptoms, enhancing bodily awareness against rumination and catastrophic beliefs, and serving as a form of mindfulness, characterized by acceptance without judgment and direct experience of physical emotions in the present moment, sometimes referred to as feeling embodiment (Aposhyan 2004; Farb et al. 2013; Price and Smith‐Dijulio 2016).

The role of body awareness in addiction has been emphasized in cognitive neuroscience models, suggesting that the foundation of awareness, reward, impulse control, and overall self‐awareness (the integration of mind and body) could be involved. Ultimately, the goal of this treatment is to improve individual functioning, attention processing, problem‐solving, and emotional regulation (Aposhyan 2004; Mischke‐Reeds 2018; Price and Hooven 2018; Price and Smith‐Dijulio 2016).

This study hypothesizes that Body Awareness Psychotherapy will significantly decrease internet addiction, boredom, and decision‐making impairments compared to a control group. By reconnecting individuals with their bodily sensations and internal states, this therapeutic approach may offer them alternative coping skills to replace compulsive internet use. It is also expected to enhance time perception and key executive functions such as impulse control, cognitive flexibility, and response inhibition. These cognitive and emotional improvements are expected to mediate the reduction in internet addiction. These executive functions are crucial for self‐regulation and are often impaired in people struggling with behavioral addictions. Improvements in impulse control could help participants resist the urge to overuse the internet, while better cognitive flexibility might allow them to consider alternative activities and responses to triggers. Stronger response inhibition could enable individuals to pause and think before automatically engaging in internet‐related behaviors. Overall, the study aims to determine whether enhancing body awareness can positively influence the cognitive‐emotional mechanisms underlying behavioral addictions like internet addiction.

## 2 Materials and Methods

### 2.1 Participants and Procedures

#### 2.1.1 Participants

**FIGURE 1:**
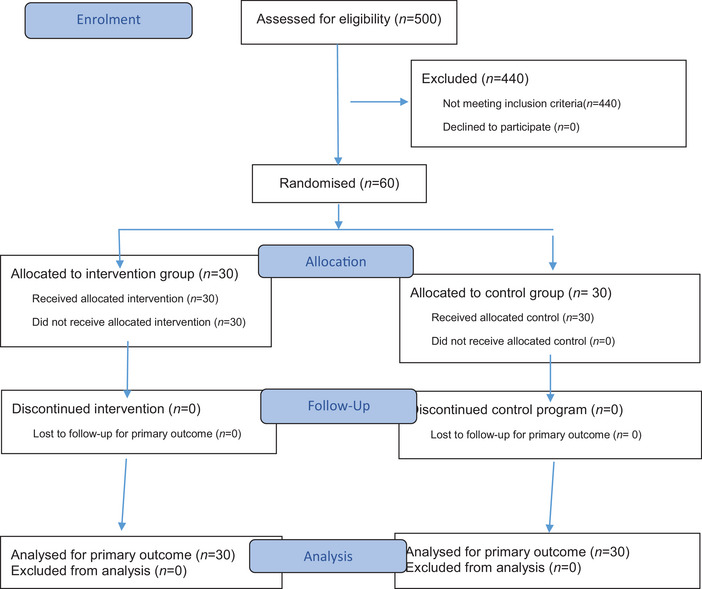
Consort flow diagram.

This study was conducted among students at Kashan University of Medical Sciences, who are considered one of the most vulnerable and high‐risk groups for internet addiction. Initially, 500 participants were screened for eligibility and enrolled in the study. A multi‐stage sampling method was used. Randomization was performed using Stat Trek software based on a random number table. A total of 60 students, diagnosed with internet addiction by scoring above 64 on Chen's test and confirmed through clinical interviews for restlessness and internet dependence, were divided into two groups of 30: an experimental group and a control group. All intervention stages were conducted after obtaining written consent from participants and under the supervision of the researcher. Participants were unaware of their group assignments, making this a single‐blind study. The experimental group received body awareness psychotherapy, while the control group participated in general psychology discussion sessions (covering the histories of psychologists) with the same number of sessions as the experimental group. The intervention lasted 60 days, as shown in Figure 1.

#### 2.1.2 Procedures

This clinical trial was assigned the ethics code IR.KAUMS.MEDNT.REC.1403.011 and is registered as clinical trial IRCT20240429061584N1. Participants were students aged 18–30 with internet addiction, confirmed by a diagnosis based on Chen's Addiction Scale, with a score above 64. After an interview conducted by the researcher, participants were informed about the procedure, and they had to meet specific conditions: no substance abuse, non‐smoking, no current or past psychological therapy, no mental disorders, and no use of psychiatric medications.

Body awareness psychotherapy was popularized by Susan Aposhyan (Aposhyan 2004). In this study, body awareness was utilized based on the book by Manuela Mischke‐Reed titled “Body Awareness Psychotherapy for Stress and Trauma” (Mischke‐Reeds 2018). The training was gradually conducted over eight sessions, each lasting 90 minutes, and held once a week. The training began with the goal of developing body literacy (the ability to identify and describe sensory awareness).

In the subsequent phase, the training included a series of body awareness exercises designed to assess and cultivate body awareness of inner emotions in various ways. Finally, a series of body awareness exercises was taught consciously, aimed at building the capacity to maintain awareness in the body. Throughout the training process and at the end of each session, participants performed a daily exercise based on their experiences during the session. The goal of the at‐home practice was to integrate internal self‐care skills into everyday life (Mischke‐Reeds 2018).

### 2.2 Therapy and Assessments

#### 2.2.1 Therapy

**Table:** 

**Sessions**	**Therapeutics aims**
1	Introductions between clients and the therapist, review of therapy course rules, self‐preparation skills, and development of self and body awareness.
2	Therapeutic attitude, experiential perspective on working with the body, facilitating bodily processes, and tracking tools.
3	Mindfulness and body awareness, reading the body, soma gram, and body roles.
4	Presence, perception, and feeling, dual awareness, and emotion regulation.
5	Movement interventions, boundaries, and body posture.
6	Gestures and non‐verbal body communication, emotion, and self‐regulation.
7	The body and self‐image, perception of time.
8	Summary and review of previous sessions, collecting feedback from clients, and discussing the application of learned concepts about internet use.

#### 2.2.2 Assessments

- *Chen Internet Addiction Scale (CIAS)*: This scale, developed by Chen and colleagues in 2003, consists of 26 items across 5 main dimensions to assess internet addiction. These five dimensions refer to lack of control, withdrawal symptoms, tolerance, interpersonal relationship problems, and health and time management. This study uses the Persian version. The validity and reliability of the questionnaire were assessed using factor analysis, internal consistency, convergent validity, and Cronbach's alpha. The convergent validity indicated *r* = 0.85 with *p* < 0.001, and the results of factor analysis using Varimax rotation indicated the extraction of five factors. In the reliability study, the results of Cronbach's alpha indicated *α* = 0.93. The CIAS Internet Addiction Assessment Questionnaire has acceptable validity and reliability (Mak et al. 2014).
- *Multidimensional state boredom scale*: This scale was designed by Fahlman in 2002. This form consists of 29 items and includes five dimensions: uniformity, high arousal, disengagement, low arousal, and time perception (inattention). This study uses Persian. The high correlation between scale items with the total score and between subscales in this study indicates the relevant internal consistency. Cronbach's alpha in the present study was estimated to be 0.92, which indicates internal consistency in Iran (Fahlman et al. 2011).
- *Emotion regulation difficulties scale*: This scale assesses individuals on a five‐point scale, ranging from 1 (rarely) to 5 (*almost always*), across six areas. The total score for an individual's difficulties in emotion regulation is calculated from the scores of these six subscales. Cronbach's alpha coefficients for questions on the denial of negative emotions ranged from 0.73 to 0.88. These coefficients confirm the internal consistency of the Persian version of the Difficulty in Emotion Regulation Scale (Gratz and Roemer 2004).
- *Time perception task*: In a computer‐based game for time perception, this study uses the Persian version to perform a specific task for a duration equal to the time the stimulus is presented. In this task, the stimulus was a black circle presented in the center of the monitor against a gray background for a short duration (400, 700, or 800 milliseconds) and a long duration (1000, 1200, or 2800 milliseconds). They were instructed to remember the duration of the circle's presence on the monitor and, immediately after the circle disappeared, to reproduce the duration by pressing and holding the space key for the same amount of time. In a 20‐ to 10‐day retest on 23 people, the reliability coefficient was 0.57 for the short period, and 0.75 for the long period (Jabarzadeh Chaharbarod et al. 2023).
- *Stroop effect task*: The Stroop test was first introduced in 1935 by John Ridley Stroop. This study uses the Persian version. In this phase of the Stroop test, participants are presented with 48 congruent color words (matching colors) and 48 incongruent color words (non‐matching colors) in red, blue, yellow, and green. Each stimulus is displayed on the screen for 2 s, and the interval between the presentations of two stimuli is 800 milliseconds. The level of inhibition or interference is calculated by subtracting the number of correct responses to incongruent words from the number of correct responses to congruent words. Additionally, a longer average response time to incongruent stimuli compared to congruent ones is another indicator for evaluating interference. The Stroop test has demonstrated good reliability and validity in measuring inhibition in adults and children. Test‐retest reliability has been reported to range from 0.80 to 0.91 (Asaadi et al. 2016).
- *The Iowa Gambling Task (IGT)*: Antonio Damasio and his colleagues held academic positions at the University of Iowa, which became known as the Iowa Gambling Task. The Iowa decision‐making task is a computer software game. This task is primarily designed to assess real‐life decision‐making, where participants are shown four card locations on a monitor. Participants are informed that each time they choose a specific card, they will win a cash prize. However, every so often, choosing a card will result in a monetary loss for them. Each card drawn will assign a reward; in some cases, a card may even incur a penalty. The original version of Beccara et al. and its computerized version have been standardized in different populations. The characteristics of the Iowa Decision‐Making Task. Studies of the standardization of this tool in Iran have shown that Cronbach's alpha is 0.89 and its split‐half coefficient is 0.84 (Salimi Nave et al. 2022).

### 2.3 Data Analysis

In this study, SPSS version 23 was used for data analysis. First, the normal distribution of the quantitative variables was determined using the Kolmogorov–Smirnov test. Demographic characteristics were presented as mean ± standard deviation (SD). Independent‐sample *t*‐tests and chi‐square tests were utilized to compare anthropometric data between groups. To examine the intervention effect, pre‐test and post‐test analyses were conducted using covariance analysis. Mediation analyses were performed using bootstrapped structural equation modeling.

## 3 Results

Note that 500 participants were initially screened for eligibility and enrolled in the study, and the Chen Internet Addiction Questionnaire was completed, and those who scored above 65 were selected. Of these projects, 60 participants were randomly assigned to either the experimental group, body awareness therapy, or the control group, which received general discussion. Note that 60 individuals were considered for the final investigation, with 30 representing the intervention group and 30 representing the control group (Figure 1).

Description of the research sample members based on demographic characteristics and determination of the status of demographic characteristics as shown in Table 1. After the central and dispersion indices of the participants' scores in the study variables are presented, the intervention has likely been effective, as shown in the pre‐test and post‐test (Tables 2 and 3). Levene's test has been used to check for homogeneity of variances, and the results of Levene's test are presented in Table 4. Since the *p*‐value is greater than the significance level, which is 0.05, the hypothesis of equal variances is upheld.

**TABLE 1:** Demographic characteristics.^a^

Variables	Control group (*n* = 30)	Intervention group (*n* = 30)	*p* value
Age (year)^b^	24.50 ± 3.10	25.07 ± 3.10	0.48
Gender (%)^c^			0.12
Male	17 (56.7)	11 (36.7)	
Female	13 (43.3)	19 (63.3)	
Education (%)^c^			0.79
Medicine	16 (53.3)	17 (56.7)	
Paramedicine	14 (46.7)	13 (43.3)	
Marital status (%)^c^			0.43
Single	16 (53.3)	19 (63.3)	
Married	14 (46.7)	11 (36.7)	

^a^ Data are percentage and mean ± SDs.

^b^ Obtained from independent *t*‐test.

^c^ Obtained from Pearson chi‐square test.

**TABLE 2:** Descriptive statistics of pre‐test scores of participants.

Variable	Group	*N*	Mean	Standard deviation	Standard error	Minimum	Maximum
**Internet addiction**	Control	30	3.244	0.535	0.138	2.04	3.95
	Experimental	30	3.464	0.559	0.144	2.18	4.20
	Overall	60	3.354	0.549	0.100	2.04	4.20
**Boredom**	Control	30	3.950	0.917	0.236	1.75	5.00
	Experimental	30	4.383	0.743	0.191	2.50	5.00
	Overall	60	4.166	0.849	0.155	1.75	5.00
**Decision making**	Control	30	4.321	0.637	0.167	1.22	5.00
	Experimental	30	3.932	0.397	0.123	1.73	5.00
	Overall	60	3.286	0.547	0.172	1.37	4.36
**Time perception**	Control	30	4.741	0.525	0.162	1.22	5.00
	Experimental	30	3.921	0.401	0.154	1.73	5.00
	Overall	60	4.331	0.542	0.167	1.37	4.50

**TABLE 3:** Descriptive statistics of post‐test scores of participants.

Variable	Group	*N*	Mean	Standard deviation	Standard error	Minimum	Maximum
Internet addiction	Control	30	3.272	0.370	0.095	2.69	3.92
	Experimental	30	3.588	0.557	0.143	2.54	4.47
	Overall	60	3.430	0.491	0.089	2.54	4.47
Boredom	Control	30	4.183	0.804	0.207	1.75	5.00
	Experimental	30	2.852	0.783	0.202	1.13	3.75
	Overall	60	3.517	1.032	0.188	1.13	5.00
Decision making	Control	30	2.971	0.671	0.168	1.13	4.67
	Experimental	30	3.471	0.379	0.213	1.27	5.00
	Overall	60	3.279	0.380	0.192	1.24	5.00
Time perception	Control	30	2.927	0.620	0.167	1.13	4.50
	Experimental	30	3.351	0.357	0.212	1.13	5.00
	Overall	60	3.139	0.345	0.189	1.13	5.00

**TABLE 4:** Levene's test output.

Variables	Levene statistic	Significance
Internet addiction	2.218	0.142
Boredom	2.167	0.133
Decision making	2.229	0.138
Time perception	3.110	0.083

The result indicates that there is a difference in internet addiction levels. It was claimed that body awareness psychotherapy is effective in influencing the levels of internet addiction. The statistical analysis between these two shows that since the significance value is < 0.05 (0.000), this is confirmed (Tables 5 and 6).

**TABLE 5:** ANOVA output of the mean analysis.

Group	Test	Mean	Standard deviation
Experimental	Internet addiction	3.321	0.752
Control		3.125	0.625
Total		3.223	0.685
Control	Boredom	4.166	0.849
Experimental		3.517	1.032
Total		3.842	0.992
Control	Decision‐making dimensions	3.297	0.752
Experimental		3.325	0.625
Total		3.311	0.685
Control	Time perception	3.321	0.752
Experimental		3.125	0.625
Total		3.223	0.685

**TABLE 6:** ANOVA test output for pre‐test and post‐test analysis: Internet addiction, boredom, decision‐making, and time perception.

	Sum of squares	Mean square	*F* statistic	Probability
**Internet addiction**				
Within group	52.709	13.177	15.748	0.000
Between group	104.591	0.837		
Total	157.300			
**Boredom level**				
Within group	35.912	8.978	7.987	0.000
Between group	140.518	1.124		
Total	176.431			
**Decision‐making**				
Within group	43.979	10.995	12.632	0.000
Between group	108.798	0.870		
Total	152.777			
**Time perception**				
Within group	43.212	10.524	12.102	0.000
Between group	102.992	0.718		
Total	146.244			

There is a significant difference in boredom levels. It was claimed that body awareness psychotherapy is effective in influencing boredom. The statistical analysis between these two indicates that, according to the significance value, which is < 0.05 (0.000), this is also confirmed (Tables 5 and 6).

There is a difference in decision‐making dimensions. To verify this result, it was claimed that body awareness psychotherapy is effective in influencing decision‐making. The statistical analysis shows the significance value is < 0.05 (0.000), and this is confirmed (Tables 5 and 6).

Body Awareness Therapy affects time perception before and after intervention, which statistical analysis between these two shows according to Tables 5 and 6. Since the significance value is < 0.05 (0.000), this hypothesis is therefore confirmed.

The research suggests that body awareness psychotherapy is effective in reducing internet addiction, with executive functions (cognitive flexibility) acting as a mediating factor. The path coefficient is outside the range of −1.96 to +1.96, indicating that this relationship is significant. Therefore, according to the decision‐making flowchart of the bootstrapping method, mediation analysis is possible. Next, the results for the model with mediating effects are displayed to examine the indirect effects. Another mediating role of executive functions (cognitive flexibility) is effective in reducing internet addiction. The indirect path and its significance level are obtained as being < 0.05, indicating that the variable in question has a mediating role. Since the influence of the mediating variable on the dependent variable is also significant, this is confirmed (Table 7).

**TABLE 7:** Regression coefficients for cognitive flexibility, impulse control, response inhibition, and emotion regulation acting as a mediating factor in the total effect model.

	Predictor	Estimate	Error	Critical ratio	Probability	Result
**Cognitive flexibility**	Psychotherapy <—Addiction	0.842	0.167	6.109	***	Confirmed
**Impulse control**	Psychotherapy <—Addiction	0.839	0.082	7.493	***	Confirmed
**Response inhibition**	Psychotherapy <—Addiction	0.839	0.082	7.493	***	Confirmed
**Emotion regulation**	Psychotherapy <—Addiction	0.669	0.129	4.319	***	Confirmed

Cognitive flexibility: 0.000, Impulse control: 0.001, Response inhibition: 0.000, Emotion regulation: 0.001

Body awareness psychotherapy is effective in reducing internet addiction, with executive functions (impulse control) acting as a mediating factor. The path coefficient is outside the range of −1.96 to +1.96, indicating that this relationship is significant. Therefore, according to the decision‐making flowchart for the bootstrapping method, mediation analysis is possible. Next, the results for the model with mediating effects are displayed to examine the indirect effects. The research indicates that body awareness psychotherapy, with the mediating role of executive functions (impulse control), is effective in reducing internet addiction. The indirect path and its significance level are obtained as being < 0.05, indicating that the variable in question has a mediating role. Since the influence of the mediating variable on the dependent variable is also significant, this is also confirmed (Table 7).

Body awareness psychotherapy is effective in reducing internet addiction, with executive functions (response inhibition) acting as a mediating factor. The path coefficient is outside the range of −1.96 to +1.96, indicating that this relationship is significant. Therefore, according to the decision‐making flowchart of the bootstrapping method, mediation analysis is possible. Next, the results for the model with mediating effects are displayed to examine the indirect effects. The research indicates that body awareness psychotherapy, with the mediating role of executive functions (response inhibition), is effective in reducing internet use. The indirect path and its significance level are obtained as being < 0.05, indicating that the variable in question has a mediating role. Since the influence of the mediating variable on the dependent variable is also significant, this hypothesis is confirmed (Table 7).

Body awareness psychotherapy is effective in reducing internet addiction, with emotion regulation acting as a mediating factor. The path coefficient is outside the range of −1.96 to +1.96, indicating that this relationship is significant. Therefore, according to the decision‐making flowchart of the bootstrapping method, mediation analysis is possible. Next, the results for the model with mediating effects are displayed to examine the indirect effects. The indirect path and its significance level are obtained as being < 0.05, indicating that the variable in question has a mediating role. Since the influence of the mediating variable on the dependent variable is also significant, this is confirmed (Table 7).

## 4 Discussion

The study identifies executive functions, decision‐making, impulse control, and response inhibition as key mediators in the relationship between body awareness psychotherapy and reductions in internet addiction. Each mediation analysis confirms significant indirect paths, indicating that these executive functions serve as important intermediaries in the therapy's effectiveness. Additionally, emotion regulation is highlighted as a mediating factor in decreasing internet addiction, also showing significant results. Overall, the findings reinforce that body awareness psychotherapy greatly impacts internet addiction levels and emphasize the role of executive functions and emotion regulation as mediators. These findings agree with previous research showing that executive function deficits are core cognitive vulnerabilities in individuals with IAD.

Recent meta‐analyses confirm that internet addiction involves disrupted reward processing and diminished executive control, particularly in the anterior cingulate and prefrontal cortices, contributing to addictive behaviors and impaired decision‐making (Fendel et al. 2024). Functional imaging in adolescence further reveals altered connectivity in executive control networks, underscoring deficits in inhibitory control, time perception, and impulse regulation commonly associated with Internet addiction (Ma et al. 2024).

Body awareness interventions that promote interoception and embodied mindfulness are increasingly recognized as valuable for addiction treatment. Interoceptive training improves understanding of somatic‐emotional cues, reduces impulsivity, and supports self‐regulation through insula‐ACC networks. Mindfulness‐based protocols have proven effective in altering neurocognitive dynamics, enhancing inhibitory control and emotional regulation, and decreasing automatic urges (Herman 2023). This body‐somatic synergy positions body psychotherapy as more than a behavioral technique; it is a psychophysiologically grounded intervention that addresses internet addiction's cognitive emotional underpinnings (Tymofiyeva et al. 2024). Meta‐evidence suggests exercise‐based and group counseling interventions improve cognition, mood, and self‐control in internet addiction (Lu et al. 2025), but combining somatic mindfulness with cognitive‐emotional training may yield stronger outcomes (Chang and Lee 2024; Lu et al. 2025). Integrating body psychotherapy provides a framework for therapies that harness embodied attention while reinforcing neurocognitive mechanisms of adaptive decision‐making and emotional resilience (Bjureberg et al. 2023).

Impairments in impulse control, cognitive flexibility, and response inhibition decrease a person's ability to manage digital behavior, leading to compulsive use patterns (Ioannidis et al. 2019; Y. Zhou et al. 2011). Our results support the idea that body awareness psychotherapy, through somatic focus and body‐based mindfulness practices, enhances higher‐level cognitive regulation by stimulating prefrontal networks involved in goal‐oriented behavior and inhibition (Farb et al. 2012; Mehling et al. 2012).

The improvement in time perception observed in this study also contributes to a growing body of evidence linking attentional regulation and internal timing mechanisms to internet overuse. Prior research has shown that individuals with IAD often display impaired temporal estimation and retrospective time judgments, which are related to attentional deficits and altered dopamine function in prefrontal‐striatal pathways (Hormes et al. 2014; Zhang et al. 2016). Body awareness psychotherapy may help recalibrate distorted time perception by increasing interceptive awareness and promoting attentional anchoring in present‐moment bodily sensations, a process consistent with the attentional gate model of time perception (Hormes et al. 2014; Ogden et al. 2011; Zhang et al. 2016).

In parallel, the role of emotion regulation as a significant mediator confirms existing models that describe IAD as a maladaptive coping mechanism for managing stress, negative affect, and boredom (Critchley and Harrison 2013; Price and Hooven 2018). Body awareness psychotherapy targets emotional self‐regulation through embodiment and present‐centered awareness, offering a bottom‐up regulatory mechanism that complements cognitive strategies. This is consistent with neuroimaging findings showing that body‐centered therapies increase functional connectivity between the anterior cingulate cortex, insula, and dorsolateral prefrontal cortex regions responsible for integrating emotional and executive functions (Eastwood et al. 2012; Farb et al. 2012).

Boredom, a frequently overlooked psychological factor in addiction models, was also significantly reduced through body awareness psychotherapy in our study. Chronic boredom has been associated with increased susceptibility to impulsive behaviors, poor attentional control, and internet overuse as a compensatory strategy (Bench and Lench 2013; Mercer‐Lynn et al. 2014). Our findings suggest that body awareness psychotherapy enhances internal stimulation and attentional engagement, helping participants tolerate low‐stimulation environments without resorting to compulsive digital behavior. This supports theoretical frameworks positing boredom as a failure of attentional self‐regulation and emotional engagement (Bench and Lench 2013; Z. Wang et al. 2020).

Moreover, the improvement in decision‐making observed through the Iowa Gambling Task reinforces the hypothesis that somatic and emotional self‐awareness enhances risk evaluation and future‐oriented choices. Internet addicted individuals often display a preference for immediate gratification and poor foresight, hallmarks of decision‐making impairments associated with prefrontal cortex dysfunction (Anderson et al. 2021; Bechara et al. 2002; Paasche et al. 2019). Body awareness psychotherapy may strengthen somatic markers, internal physiological signals associated with affective responses, which guide individuals toward more adaptive decision‐making, as proposed by Damasio's somatic marker hypothesis (Aposhyan 2004; Price and Hooven 2018; Price and Smith‐Dijulio 2016).

Collectively, these results align with dual‐process models of addiction that emphasize the imbalance between impulsive, emotionally driven responses (bottom‐up) and controlled, deliberate cognitive regulation (top‐down) (Cerniglia et al. 2020; Nuyens et al. 2019; Y. Wang et al. 2019; Wegmann et al. 2018). Body awareness psychotherapy appears to influence both pathways by integrating interoceptive, attentional, and executive mechanisms, offering a holistic therapeutic modality for addressing behavioral addictions such as IAD (González‐Bueso et al. 2018; Y. Wang et al. 2019).

This study has several limitations. First, the sample was limited to university students, which may restrict the generalizability of the findings to broader or clinical populations. Second, the intervention period was limited to 8 weeks, and no follow‐up data were collected to assess long‐term effects. Third, although cognitive and emotional mechanisms were measured behaviorally, no neurophysiological data were collected to verify neural correlates. Future studies could incorporate neuroimaging, longer‐term follow‐up, and comparisons with other therapeutic approaches, such as cognitive‐behavioral therapy (CBT) or mindfulness‐based cognitive therapy (MBCT), to evaluate the specific contribution of somatic awareness in addiction treatment.

## 5 Conclusion

The present study provides compelling evidence that body awareness psychotherapy is an effective intervention for reducing IAD by targeting both cognitive and emotional regulatory mechanisms. Body awareness psychotherapy significantly improved executive functions, particularly cognitive flexibility, impulse control, and response inhibition, as well as emotion regulation. These enhancements mediated reductions in internet addiction severity, boredom, and maladaptive decision‐making patterns. Furthermore, improvements in time perception suggest that body awareness psychotherapy may recalibrate attentional and interoceptive processes disrupted by chronic digital overuse. As a low‐cost, non‐pharmacological, and body–mind integrative approach, body awareness psychotherapy holds promise as a novel intervention for behavioral addictions, warranting further research in diverse populations and neurobiological contexts.

## Author Contributions

**Maliheh Fallah**: conceptualization, methodology, software, data curation, validation, investigation, writing–original draft, funding acquisition, writing–review and editing, visualization, formal analysis, project administration, resources, supervision. **Hamidreza Mohajerani**: conceptualization, methodology, software, data curation, validation, investigation, writing–original draft. **Abdullah Omidi**: conceptualization, methodology, validation, investigation, visualization, project administration, writing–original draft. **Amir Ghaderi**: conceptualization, methodology, software, data curation; supervision, project administration, visualization, funding acquisition, investigation, validation, writing–review and editing, writing–original draft.

## Conflicts of Interest

The authors declare no conflicts interest.

## Peer Review

The peer review history for this article is available at https://publons.com/publon/10.1002/brb3.70846

## Ethics Statement

The study design and protocol were approved by the Kashan University of Medical Sciences' ethics committee, and the study was conducted by the Declaration of Helsinki IR.KAUMS.MEDNT.REC.1403.011. Written informed consent was signed by each participant. The study is listed in the Iranian Clinical Trials Registry (IRCT20240429061584N1). After being approved by the relevant ethical review committee, protocol changes to the clinical protocol will be updated to the clinical trials registry.

## Data Availability

All data and supporting information can be obtained from the respective author upon a reasonable request.
